# The Clinical Significance and Mechanisms of REG4 in Human Cancers

**DOI:** 10.3389/fonc.2020.559230

**Published:** 2021-01-08

**Authors:** Junyan Zhang, Zhi Zhu, Zhifeng Miao, Xuanzhang Huang, Zhe Sun, Huimian Xu, Zhenning Wang

**Affiliations:** Department of Surgical Oncology and General Surgery, Key Laboratory of Precision Diagnosis and Treatment of Gastrointestinal Tumors, Ministry of Education, The First Affiliated Hospital of China Medical University, Shenyang, China

**Keywords:** REG4, cancer, mechanism, clinical significance, biological function

## Abstract

Regenerating islet-derived type 4 (*REG4*), a member of the calcium-dependent lectin gene superfamily, is abnormally expressed in various cancers, such as colorectal, gastric, gallbladder, pancreatic, ovarian, prostate, and lung cancer. *REG4* is associated with a relatively unfavorable prognosis and clinicopathologic features in cancers, including advanced tumor and nodal stage, histological differentiation, and liver and peritoneal metastasis. Moreover, *REG4*-positive cancer cells show more frequent resistance to chemoradiotherapy, especially 5-FU-based chemotherapy. *REG4* participates in many aspects of carcinogenesis, including cell proliferation, apoptosis, cell cycle, invasion, metastasis, and drug resistance. The underlying mechanisms are complex and involve a series of signaling mediators and multiple pathways. Thus, *REG4* may be a potential diagnostic and prognostic biomarker as well as a candidate therapeutic target in cancer patients. In this review, we systematically summarize the advances about the clinical significance, biological functions, and mechanisms underlying *REG4* in cancer to provide new directions for future cancer research.

## Introduction

The regenerating islet-derived (*REG*) family genes belong to the calcium-dependent lectin (C-type lectin) gene superfamily. There are five REG members expressed in humans: REG1A, REG1B, REG3A, REG3G, and REG4. All of these are located on the second chromosome, except *REG4*, which is located on chromosome 1 (1). REG members are identified to be essential for cell proliferation, regeneration, inflammation, tumor formation, and formation of immune system (1). Of these, *REG4* is the most frequently observed member and has been characterized as a key regulator in the initiation, differentiation, and progression of various human cancer cell types.

*REG4* was originally identified by a high-throughput sequence analysis of a cDNA library derived from patients with inflammatory bowel disease (2). It is located on the long arm of chromosome 1, contains six introns and seven exons, and encodes 158 amino acids that include a signal peptide of 22 amino acids and a conserved calcium-dependent hydrocarbon recognition domain (CRD). CRD is located at amino acid positions 30–155 in the REG4 protein and is critical for the biological function of REG4, especially in its promotion of invasion and migration abilities (3). Unlike other C-type lectins, REG4, in the absence of calcium, can bind heparin, polysaccharides and mannan mediated by CRDs and shows a potential role in specific carbohydrate recognition (4). These findings may provide clues to understanding the molecular interactions with currently uncertain receptors and the sugar-binding role of REG4 protein.

REG4, a small secretory protein sized about 18-kD, is also referred to as regenerating protein-like protein (RELP) (5). *REG4* is expressed in parietal cells of the gastric mucosa and epithelial neuroendocrine cells of the small intestine (5, 6), and inflammatory bowel disease (6–9). REG4 may be involved in the metaplastic responses and inflammation of the gastrointestinal epithelium. The expression levels in cancerous tissues, such as the stomach, pancreatic, colorectal, prostate, gallbladder, ovarian and lung cancers are much higher than that in normal tissues (6, 9–13). As a secretory protein, REG4 shows two mucin-like and perinuclear patterns with immunohistochemical staining (14) and promotes carcinogenesis in tumor cells *via* both autocrine and paracrine manners (15). The expression of REG4 was associated with clinical characteristics, such as histologic differentiation, invasion depth, and TNM stage in cancer patients and is recommended to be a promising biomarker for predicting metastasis, combined with S100A4 and MACC1 (16). The combination of VEGF-C and REG4 has been characterized as a promising factor for clinical staging to supplement the TNM classification system (17). High expression of REG4 predicts poor prognosis and drug-resistance by promoting cancer cell proliferation, invasion and anti-apoptosis (18).

Kumar et al. reported that REG4 promotes cell proliferation in colon adenocarcinoma cells *via* the EGFR/Akt/AP-1 pathway (19). The mechanisms involved are far more complex than perceived. The understanding of mechanisms of REG4 in many cancer types has increased in the recent years (**Tables 1** and **2**). The current review will focus on the clinical significance and underlying mechanisms of *REG4* in various human cancers and highlight its potential applicability for diagnostic, prognostic and therapeutic approaches.

**Table 1:** The role and clinical significance of REG4 in human cancers.

Cancer type	Results	Reference
Colorectal	Associated with aggressive phenotype, unfavorable clinical parameters such as advanced tumor and nodal status, also the drug-resistance	(5, 8, 20, 21)
cancer	Upregulated in adenomas with dysplasia, inflamed epithelium.	(22, 23)
	Favorable clinical parameters and favorable prognosis for non-mucinous colorectal cancers.	(24)
	Serum level increased in patients with liver metastasis	(25)
	Increased resistance to IR-induced apoptosis.	(8)
	Also expressed in in the neoplastic goblet cells of appendiceal mucinous cystadenomas and epithelial implants of pseudomyxoma peritonei	(26)
	Promote proliferation and resistance to apoptosis	(8, 9, 19, 27),
	Associated with advanced T and N status, poor therapeutic response and poor prognosis for neoadjuvant CCRT in rectal cancer patients.	(20, 28)
Gastric cancer	Associated with intestinal and neuroendocrine differentiation of gastric carcinoma, expressed in the goblet cells of intestinal metaplasia and neuroendocrine cells at the base of intestinal metaplasia; signet ring cell carcinoma more frequently expressed.	(6, 29, 30)
	Highly expressed in peritoneum-metastasis cases than in negative cases; promote peritoneal metastasis.	(31)
	Showed more frequently neuroendocrine differentiation: serotonin, somatostatin; coexpressed with gastrin, serotonin and pancreatic polypeptide.	(32)
	Correlated with advanced stage; Predicts poor prognosis	(33, 34)
	Serum levels elevated	(34)
	Serum level predicts resistance to 5-Fu-based chemotherapy, involved in apoptosis-related genes Bcl-2 and dihydropyrimidine dehydrogenase.	(18, 33)
Pancreatic	Serum level or tissue protein elevated in pancreatic cancer	(35–37)
cancer	Elevated in intestinal-type IPMNs	(11)
	REG4-overexpressing cancer cells resistant to chemoradiotherapy and more frequently local recurrence	(38, 39)
	Pro-proliferation	(40)
	Not independent prognostic factor; Just used in differential diagnosis between pancreatic malignant cancer and chronic pancreatitis	(41)
Ovarian cancer	Elevated in cancer tissues, especially mucinous carcinomas, intestinal-type; REG4 expression was enhanced by transfection of CDX2	(42–44)
	Higher expression was observed in well-and moderately differentiated carcinomas than poorly differentiated carcinomas	(45)
	Poor prognosis	(45)
	Inhibited cell apoptosis, enhanced G_2_/S progression, proliferation, migration and invasion	(45)
Prostate	Independent prognostic indicator of relapse after radical prostatectomy	(46)
cancer	Candidate marker for hormone refractory metastatic prostate cancer	(9)
Gallbladder carcinoma	More frequently expressed in well to moderately differentiated than in poorly differentiated caners; Involved in carcinogenesis through intestinal metaplasia; Favorable prognosis	(12)
	Elevated in cancer tissues	(12, 47)
	Lower expression in well-differentiated adenocarcinoma; Poor prognosis	(47)
Salivary grands	Expressed in adenoid cystic carcinomas but not in oral squamous cell carcinomas.	(48)
Lung cancer	Elevated in KRAS mutant lung adenocarcinoma with low expression of TTF-1; Seliencing *REG4* reduced cancer cell proliferation and tumorigenesis *via* blocking G2/M transition.	(13)

IR, irradiation; IPMNs, intraductal papillary mucinous neoplasms of the pancreas; TTF-1, transcription termination factor 1.

**Table 2:** The mechanisms of REG4 involved in human cancers.

Cancer type	Related pathway or molecules	Reference
Colon adenocarcinoma	REG4 activates EGFR/Akt/AP1 pathway and downstream genes (Bcl-2 Bcl-XL, survivin and MMPs), changes.	(19)
Pancreatic cancer	REG4 promotes the polarization macrophages to M2 phenotype relying on EGFR/AKT/CREB pathway.	(49)
Colorectal cancer	REG4 promotes colorectal cancer cell division through Akt/GSK-3*β*/*β*-catenin/TCF-4 pathway.	(50)
Colorectal cancer	MiR-363 downregulates REG4 *via* suppressing GATA6 and promotes cancer cells growth.	(51)
Prostate cancer	ADAM9 induced REG4 expression indirectly and upregulated p21 level which negatively regulates Cyclin D1 and blocks G1/S transition.	(52)
Gastric cancer	GPR37 is identified as an interactive partner of REG4; positive feedback loop triggered by REG4 and consisting of GPR37, ADAM17, TGF-a, EGFR, SP1 and REG4.	(21)
Colon and prostate cancer	REG4 modulates multiple RTK activation and downstream factors, such as Hsp27, Bcl-2, p21, p27.	(53)
Gastric cancer	REG4 enhanced 5-FU-based resistance through MAPK/Erk/Bim pathway.	(54)
Cancer Stem cell	REG4 promotes cancer stem cells properties *via* Wnt/β-catenin pathway.	(55)

## REG4 Expression Pattern and Clinical Significance in Human Cancers

### Colorectal Cancer

REG4 is expressed in colorectal adenomas with dysplasia (22) or inflamed epithelium (23). Xiao et al. explored the physiological functions of REG4 in intestinal inflammation and found that REG4 altered the colonic bacterial composition and reduced the number of the bacteria adhering to the colonic epithelium *in vivo* and promoted the growth of colonic organoids *via* activation of signal transducer and activator of transcription 3 (STAT3) *in vitro* (56). REG4 was upregulated in colorectal cancer tissues than in adjacent normal mucosa (7, 10), indicating that *REG4* overexpression may be an early event in colorectal carcinogenesis. Kukka et al. also observed robust expression of REG4 in the epithelial implants of pseudomyxoma peritonei and neoplastic goblet cells of appendiceal mucinous cystadenomas (26). *REG4* overexpression is frequently associated with aggressive phenotypes, unfavorable clinical parameters such as advanced tumor and nodal status, and drug-resistance (5, 8, 20, 21). Moreover, REG4 was useful in predicting response to neoadjuvant chemoradiotherapy in patients with rectal cancer (20, 28). Kumar et al. identified a relationship of REG4 with the increased resistance to irradiation-induced apoptosis (8). Kobunai et al. found that *REG4* gene expression was 12-fold higher in radioresistant cells and might be a useful predictor of the sensitivity of rectal cancer patients to radiotherapy (57). Additionally, colorectal cancer patients with metastatic recurrence in the liver showed more frequent REG4 immunostaining and serum levels than in those without recurrence. Serum REG4 levels can be used to predict liver recurrence (25). Survival analysis revealed that high REG4 expression could be correlated with shortened survival time and emerged as an adverse prognostic factor (13, 45). Jared et al. showed that REG4-postive tumors, but not at a high risk of recurrence, were associated with decreased survival in established recurrent colon adenocarcinoma, possibly *via* activation of REG4-CD44/CD44ICD pathway (58). The above evidence indicates that *REG4* may be a potential therapeutic target in colorectal cancer. However, Kaprio et al. performed immunohistochemistry analysis in 840 consecutive surgically treated colorectal cancer patients and found that REG4 expression was associated with favorable clinicopathological characteristics. REG4 expression indicates higher overall survival rates in non-mucinous colorectal cancer patients (24). Whereas, studies have suggested that *REG4* can promote colorectal cancer cell proliferation and elevate resistance to drug-induced apoptosis, *in vivo* and *in vitro* (8, 9, 19, 27). The conflicting results may be attributed to the different cancer phenotypes included in the study or the use of different methods to measure RNA or protein levels, which may result in varied conclusions.

### Gastric Cancer

The expression of REG4 is elevated in goblet cells of intestinal metaplasia and neuroendocrine cells at the base of intestinal metaplasia (6). Zheng et al. showed that REG4 mRNA or protein expression was upregulated in the intestinal metaplasia and adenoma than in paired normal mucosa (29). Signet ring cell carcinoma, an aggressive phenotype of gastric cancer, expressed more REG4 than other types of gastric cancer (29, 30). Another study reported that REG4-positive cases showed more frequent neuroendocrine differentiation than REG4-negative cases. Double immunofluorescence staining revealed REG4 may be co-expressed with gastrin, serotonin and pancreatic polypeptide, and REG4-positive cells expressed more neuroendocrine hormones than REG4-negative cells (32). These results suggest that *REG4* plays an important role in intestinal metaplasia and neuroendocrine differentiation.

REG4 expression in gastric cancer positively correlates with the cell invasive depth, clinical stages, diffuse type, poor differentiation, distant metastasis and intrinsic drug resistance to 5-FU (33, 34). Moreover, REG4 positivity in metastasized human gastric cancer was significantly higher than that in negative cases (31). REG4-positive group showed significantly less survival time than REG4-negative group (34). Zheng et al. also reported that the serum levels of REG4 in gastric carcinoma patients were significantly higher than those in healthy individuals. Additionally, REG4 may be a better serum marker than carbohydrate antigen 19-9 (CA199) and carcinoembryonic antigen (CEA) for early diagnosis and as a prognostic indicator of gastric cancer (34). Patients with high serum REG4 level were less sensitive to 5-FU-based chemotherapy, possibly due to REG4-induced Bcl-2 and dihydropyrimidine dehydrogenase (18, 33). Zheng et al. showed that as the protein expression of REG4 in intestinal metaplasia, adenoma, carcinoma and gastritis gradually decreased according to combined immunohistochemistry and *in situ* hybridization on tissue microarray, indicates that *REG4* may be suitable to distinguish gastric benign disease and malignant tumors (29).

REG4 expression upregulates SRY-box transcription factor 9 (SOX9) and promotes invasiveness and migration in gastric tumor cells (59). Kuniyasu et al. observed increased number and size of peritoneal tumors and decreased apoptosis *in vitro*, along with worsened mice survival after transfection with REG4 (31). Antibody against REG4 significantly inhibited proliferation in gastric cancer cells (MKN45 and AGS) and synergistically enhanced the lethal effect of 5-FU *via* the MAPK/ERK/Bim pathway (54, 60). Zhou et al. also revealed that knockdown of *REG4* decreased stemness properties in gastric cancer stem cells and increased the effectiveness of cell death following chemoradiation treatment, indicating that the inhibition of endogenous REG4 may be a promising therapeutic strategy in human gastric cancer (61).

### Pancreatic Cancer

*REG4* is overexpressed in pancreatic cancer tissues than in adjacent normal tissues at either the mRNA or protein level (35–37). Kohei et al. found that intestinal-type intraductal papillary mucinous neoplasms of the pancreas (IPMNs) showed frequent moderate and severe dysplasia. Of the 125 IPMNs, 43 (34%) were positive for REG4 and almost all of the intestinal-type IPMNs (35/38) expressed REG4, suggesting that *REG4* was involved in the ‘intestinal’ carcinogenesis pathway in IPMNs (11). Serum REG4 levels could be correlated with REG4 expression in cancer tissues, and they were elevated in patients with pancreatic cancer than in healthy individuals and those with chronic pancreatitis (35, 41). Patients with higher REG4 levels showed unfavorable histologic response to chemoradiation and experienced more frequent local recurrence postoperatively (38, 39). Akio et al. found that knockdown of *REG4* resulted in a significant decrease in cell viability in pancreatic ductal adenocarcinoma. Conversely, treatment with recombinant REG4 enhanced cell growth in a dose-dependent manner, indicating that targeting *REG4* may be a potential targeted therapy in pancreatic cancer (40). A 2018 revealed that REG4 was not independent prognostic factor by multivariate analysis, although serum REG4 levels could be used in the differential diagnosis of pancreatic malignant cancers and chronic pancreatitis (41).

### Tumor of Reproductive System

REG4 is frequently expressed in mucinous ovarian cancer subtype (42, 43), especially intestinal-type, and is absent in the endocervical-like form (44). Higher expression was observed in well- and moderately- differentiated than poorly-differentiated carcinomas (45). *REG4* plays an essential role in early ovarian carcinogenesis and is closely linked with mucinous ovarian carcinomas, histologic differentiation and adverse prognosis (45). REG4, with cytokeratin (CK) 7, contributes to the differential diagnosis between primary and metastatic ovarian mucinous carcinomas (44). *REG4* overexpression and treatment with recombinant REG4 both inhibited apoptosis, and enhanced G2/S progression, cell proliferation, migration and invasion in SKOV3 ovarian cancer cells (45).

There are only two studies about the clinical role of REG4 in prostate cancer. Shinya et al. demonstrated that high expression of REG4 predicts relapse risk after radical prostatectomy (46). Another study revealed that REG4 is overexpressed in prostate tumors after neoadjuvant hormone ablation therapy, especially in hormone-refractory metastatic prostate cancer tissues (9). Moreover, high expression of REG4 in prostate cancer correlated with tumor recurrence, metastasis and therapy failure.

### Some Other Cancer Types

There are also studies revealing *REG4* overexpression in gallbladder adenocarcinomas (12, 47). However, the role and clinical significance of these findings in different studies are controversial. Yang et al. analyzed 108 gallbladder adenocarcinomas samples using immunohistochemical analysis and elucidated that the frequency of REG4-positive cases is lower in well-differentiated adenocarcinoma and that high expression predicts poor prognosis (12). Hidehiko et al. analyzed the mRNA and protein levels in 31 gallbladder carcinoma samples using quantitative reverse transcription-polymerase chain reaction and immunohistochemical staining, and demonstrated that REG4 expression was more frequent in well- and moderately differentiated than in poorly differentiated gallbladder adenocarcinoma samples. REG4 expression in gallbladder adenocarcinoma is associated with a relatively favorable prognosis in patients after surgery (47). However, elucidating the exact role in gallbladder carcinoma requires comprehensive analysis of in a larger cohort.

Further, Sun et al. analyzed 55 clinical samples and combined GEO and TCGA database information, and found that both mRNA and protein levels of REG4 were significantly upregulated in KRAS mutant lung adenocarcinoma samples with low expression of the transcription termination factor 1 (TTF-1) (identified as the KS subgroup). REG4 promotes the progression in KRAS mutant lung adenocarcinoma cells progression and can be used as a novel biomarker in lung adenocarcinoma subtype (13). Another study also reported overexpression of REG4 in invasive mucinous lung adenocarcinoma of gastric differentiation-type (62).

Finally, REG4 was also found to be expressed in adenoid cystic carcinomas in the salivary gland (17/41), but not in oral squamous cell carcinomas. The expression of REG4 could be correlated with nodal metastasis, poor prognosis, and pEGFR levels and that cell growth could be inhibited by anti-REG4 treatment *in vitro* (48).

## Mechanisms Involved in Human Cancers

### Promoting Proliferation and Resistance to Apoptosis

Overexpression and oncogenic role of epidermal growth factor receptor (EGFR) in malignant tumors are commonly identified (63, 64). The activator protein-1 (AP-1) complex, which is predominantly composed of proteins in the Jun and Fos families, is one of the most important transcription factors triggered by EGFR signaling (65). Akt is reported to be a specific upstream kinase regulating AP-1 transcription activity (66, 67). Bishnupuri et al. revealed that REG4 activates EGFR/Akt/AP-1 pathway and contributes to the increased invasiveness and resistance to apoptotic cell death in colon adenocarcinomas. Treatment with recombinant REG4 induced a remarkable increase in the phosphorylation of EGFR at Tyr992 and Tyr1068 and the activation of downstream Akt at Thr308 and Ser473, coupled with increased AP-1 transcriptional activity: quantitative increase in expression of Jun B, Jun D, and Fos B (19). Furthermore, the expression of their downstream anti-apoptotic genes (Bcl-2, Bcl-XL, survivin, and MMPs) was significantly increased (19, 68). Huang et al. also reported that REG4 promotes cell proliferation and migration in gastric cancer *via* activation of Akt (69).

REG4 can also promote cancer cell proliferation and anti-apoptosis *via* other mechanisms. Kathryn et al. revealed that REG4 can modulate phosphorylation of multiple additional receptor tyrosine kinases (RTKs), including insulin receptor, insulin-like growth factor receptor, as well as their downstream effectors, EGFR, mitogen-activated protein kinase, and phosphatidylinositol-3-kinase pathways. Knockdown of *REG4* affects the ability of insulin and EGF to phosphorylate downstream tyrosine kinase in human colon and prostate cancer cells (53). Jin et al. revealed that REG4 inhibits apoptosis by regulating the MAPK/ERK/Bim signaling pathway, thereby enhancing resistance of gastric cancer cells to 5-FU, based on the western blotting results (54). However, the precise mechanism by which REG4 mediates the phosphorylation of other RTKs and their downstream proteins and the precise role of REG4 in the MAPK pathway is still unclear and requires further research.

### Involved in Cell Cycle Regulation

**Figure 1:**
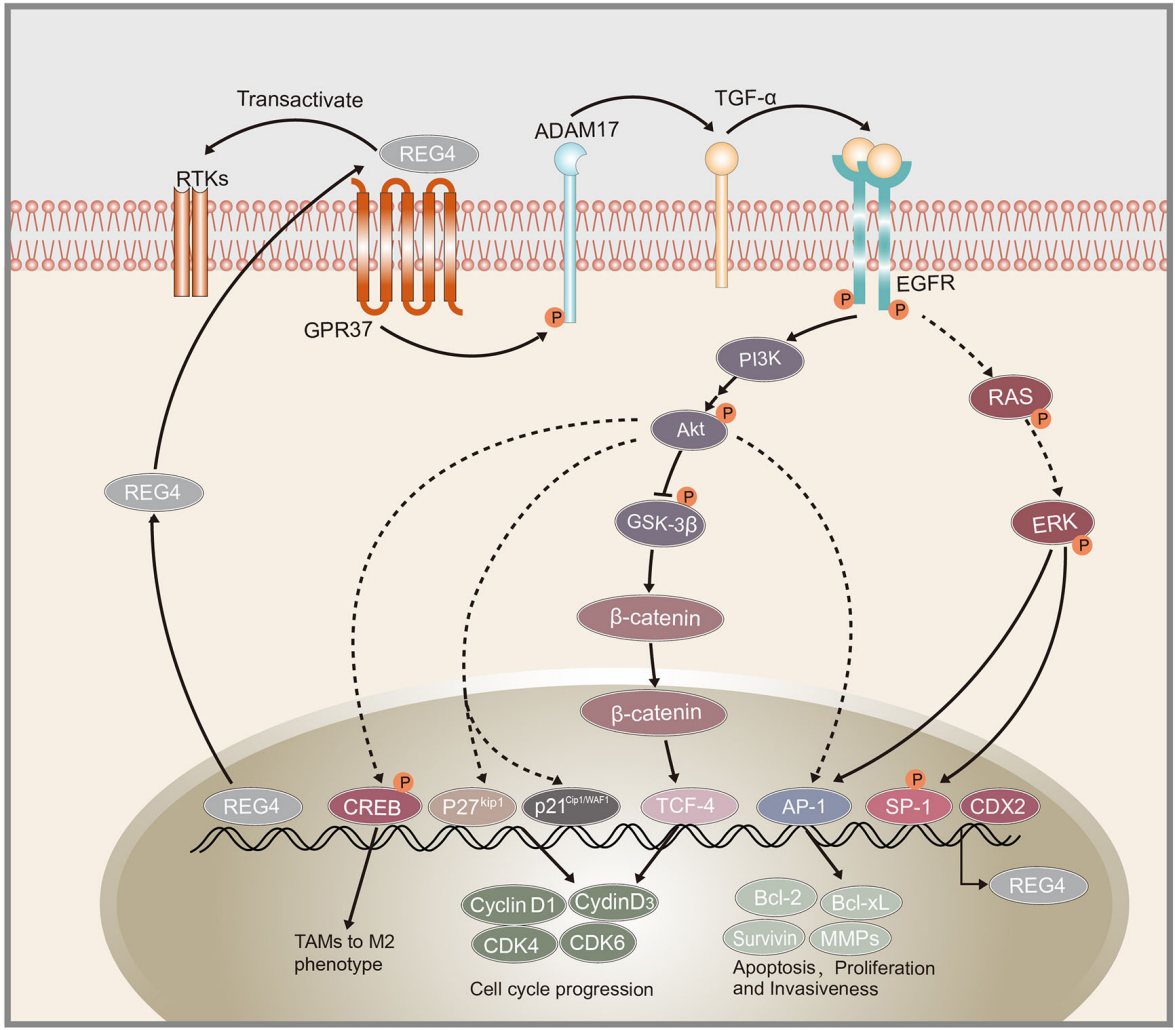
Schematic representation of REG4 signaling pathway. GPR37 as the interactive partner of REG4 complex.REG4 can transactivate RTKs including EGFR. EGFR phosphorylates Akt and activates downstream AP-1, GSK-3*β*/*β*-catenin/TCF-4, p21^Cip1/WAF1^/p27^Kip1^ pathway regulating cancer cells apoptosis, proliferation and invasiveness. EGFR and Akt can also induced the phosphorylation of CREB and promote TAMs polarization to M2 phenotype. REG4 can amplify itself by a positive feedback loop consisting of GPR37, ADAM17, TGF-α, EGFR, SP1 and REG4. CDX2 was identified as the transcription factor of REG4. REG4, Regenerating islet-derived type 4; GPR37, G protein-coupled receptor 37; RTKs, receptor tyrosine kinases; EGFR, Epidermal growth factor receptor; Akt, serine/threonine kinase 1; AP-1, activator protein-1; GSK-3β, glycogen synthase kinase 3 beta; TCF-4, transcription factor 4; CREB, cAMP response element-binding protein; TAMs, tumor-associated macrophages; ADAM17, a disintegrin and metallopeptidase domain 17; TCF-α,transforming growth factor alpha; CDX2, caudal type homeobox 2.

Growth and development of cancer depends on the ability of cancer cells to escape the normal controls and check points of cell division cycle. The division of mammalian cells is mainly regulated at specific points in the cell cycle, particularly at the G1/S and G2/M transitions. Mammalian D-type cyclins and associated cyclin-dependent kinases (CDKs) are essential for driving each cell cycle phase. Misregulated CDKs induce unscheduled proliferation and chromosomal and genomic instability (70). Furthermore, REG4 mediates increased Akt kinase activity and inactivates glycogen synthase kinase 3*β* (GSK-3*β*) by increasing phosphorylation of Ser9 residue. Decreased GSK-3*β* activity induces an increased nuclear translocation of *β*-catenin by decreasing its phosphorylation at Ser33/37/Thr41 and sequentially increasing TCF-4 transcriptional activity, which promotes the expression of cyclin D1 and D3 coupled with CDK4 and CDK6. REG4 treatment accelerates G1/S and G2/M phase transition, coupled with increased mitotic index of colorectal cancer cells. The use of REG4 antagonists or Akt inhibitors decreased, while GSK-3*β* antagonist significantly increased mitotic index and proliferation in colorectal cancer cells (50). These results indicated the key role of REG4 in regulating colorectal cancer cell division *via* the Akt/GSK-3*β*/*β*-catenin/TCF-4 signaling pathway (**Figure 1**). Moreover, the mechanism by which REG4 mediates Akt kinase activity may be attributed to the REG4-mediated phosphorylation of EGFR, as mentioned above.

Mutations in both adenomatous polyposis coli (*APC*) and *KRAS* synergistically increase tumorigenesis and enhance the induction of colorectal stem cells (71). As per the microassay-based transcriptional analysis and knockout of all the representative *KRAS*-inducible genes, knockout of *REG4* showed the most significant reduction in spheroid-forming capability in stem cells harboring mutations in both KRAS and APC. Expression of REG4 was significantly upregulated in a mutant KRAS-dependent manner in both colorectal stem cells and cancer tissues harboring APC mutation, consistent with another study with REG4 overexpression in KRAS mutant lung adenocarcinoma (13). Protein levels of p-LRP6, *β*-catenin, and p-GSK-3*β* were increased upon treatment with recombinant REG4 in a dose-dependent manner. REG4-induced activation of the GSK-3*β*/*β*-catenin signaling pathway promotes colorectal stem cell properties induced by KRAS mutation with loss of APC (55). Another study also indicated that targeting REG4 in aldehyde dehydrogenase 1 (ALDH1) positive cancer-initiating cells regulates the tumorigenic capacity of diffuse-type gastric carcinoma-initiating cells inhibited by GSK-3β (72). Moreover, REG4 was also upregulated in KRAS-mutant lung carcinoma and thus, is a novel biomarker in the lung adenocarcinoma subtype. Silencing REG4 reduced cancer cell proliferation and tumorigenesis *in vivo* and *in vitro* by blocking G2/M transition (13), suggesting an important role of REG4 in KRAS-driven lung cancer pathogenesis. However, further studies are needed to clarify the role and underlying mechanisms of REG4 in cell proliferation and division and its potential therapeutic value in lung cancer.

A disintegrin and metalloproteinase 9 (*ADAM9*) encoded protein regulates prostate cancer proliferation and invasion by interacting with a variety of cell surface proteins in prostate cancer (73–75). Expression of ADAM9 correlates with poor prognosis, recurrence risk and therapy-resistance (75, 76). Radioactive and chemical pharmaceutics or the tumor microenvironment itself can induce endogenous oxidative responses which induce ADAM9 expression (76). Liu et al. found that knockdown of ADAM9 decreases expression of REG4 and upregulates expression of p21^Cip1/WAF1^ and p27^Kip1^ which negatively regulates the expression of cyclin D1 and blocks the G1/S transition (52). Radiochemotherapy could induce the endogenous superoxide and upregulation of ADAM, followed by activation of REG4/p21^Cip1/WAF1^ pathway activation. The ADAM9/REG4/p21^Cip1/WAF1^ pathway contributes to cancer cell division and drug resistance. Furthermore, Liu et al. also reported that ADAM9 may indirectly induce REG4 expression *via* activation of EGFR by cleaving HB-EGF (52). Further investigation of the correlation between ADAM9 and REG4 may help to understand the underlying mechanism of therapy-resistance in prostate cancer. Additionally, Wang et al. revealed that REG4 promotes the phosphorylation of ADAM17 and amplifies itself *via* a positive feedback (21) which indicates that ADAM family members may be involved in the progression of REG4-induced pathological changes.

### Promoting the Polarization Macrophages to M2 Phenotype

Another study demonstrated that REG4-induced EGFR/Akt pathway activation promotes cancer cell progression directly and polarization of macrophages to M2 phenotype. Several reports suggest that M2 tumor-associated macrophages (TAMs) can provide a favorable microenvironment to promote tumor angiogenesis, progression and suppress adaptive immunity (77–79). Ma et al. demonstrated that treatment with recombinant REG4 and the culture medium of *REG4*-positive pancreatic cancer cells induced the expression of some M2-related genes in macrophages, such as IL10 and CD163 (49). TAMs are often recruited to tumors by growth factors or chemokines produced by tumor cells themselves (80). EGFR and cAMP response element-binding protein (CREB) are reported to contribute to M2 polarization of macrophages (81). Further study showed that overexpression of REG4 promotes phosphorylation-mediated activation of EGFR and Akt, which subsequently induce the phosphorylation of CREB at Ser133. However, knockdown of CREB blocked the M2 macrophage polarization mediated by REG4 (49). Tumor-secreted REG4 can change the tumor microenvironment to facilitate cancer cell growth and metastasis by promoting macrophage polarization to M2 *via* activation of the EGFR/Akt/CREB pathway.

### Molecules Regulating the Expression of REG4

The receptor of REG4 is always a problem that has been confused by researchers. Wang et al. demonstrated a positive feedback loop triggered by REG4, amplifying itself *via* EGFR, comprising EGFR, ADAM17, G protein-coupled receptor 37 (GPR37), TGF-α, REG4, and transcription factor SP1 (21), as shown in **Figure 1**. They also demonstrated that GPR37 is a partner of REG4 and promotes peritoneal metastasis in gastric cancer cells by mediating the signal transduction of REG4 (21). However, there is still no study elucidating the exact receptor or the complete complex partners of REG4. Apichat et al. also showed that the expression of *REG4* in colon cancer cells can be enhanced by stimulation from transforming growth factor-α (TGF-α), epidermal growth factor (EGF), fibroblast growth factor, and hepatocyte growth factor (23).

The glutamyl-tRNA amidotransferase (*GATA*) family, a group of evolutionarily conserved zinc finger-containing transcription factors, is essential for proliferation, differentiation and development in many organs (82). Among them, *GATA6* is expressed throughout the gastrointestinal epithelium and is essential for the tumorigenicity and cell invasion in colorectal cancer (83). Yoshihiro et al. showed that miR-363 represses transcription of REG4 *via* suppression of GATA6. GATA6 simultaneously induces expression of leucine-rich repeat containing G-protein-coupled receptor 5 (LGR5) and is presented as a stem cell marker (84, 85). Cooperation between the GATA6/LGR5 and GATA6/REG4 pathways plays an important role in the tumorigenicity in colon cancer cells (51). Yoshihiro et al. also reported that the expression levels of REG4 and LGR5 may not be directly influenced by miR-363 and GATA6. GATA6 usually acts in combination with other transcriptional factors, including TCF-4 and caudal type homeobox 2 (CDX2) (86, 87). CDX2 was frequently found to bind directly to the 5′-flanking promoter of *REG4* and positively regulate its expression (11, 42, 44, 88, 89). CDX2 may be involved in the process of inducing upregulation of REG4 *via* miR-363 and GATA6, which needs further research.

Another study revealed that miR-24 directly downregulated REG4 expression by binding its 3′ untranslated region and restrained gastric cancer progression (90). Moreover, gliotactin (GLI), a transcription factor in the hedgehog signaling pathway, was also identified to bond to *REG4* promoter region and induce REG4 expression in pancreatic cancer (36).

## Conclusion and Perspective

*REG4* is upregulated not only in various human cancers, including colorectal, gastric, pancreatic, ovarian, prostate, gallbladder, and lung cancer (**Table 1**), but also in some benign diseases, such as ulcerative colitis, intestinal metaplasia, adenoma, and atypical hyperplasia, suggesting a significant role of REG4 in tumorigenesis. Most studies have revealed that *REG4* overexpression is positively associated with unfavorable clinical parameters, resistance to therapy and poor prognosis, indicating that *REG4* is a promising prognostic biomarker and potential therapeutic target in cancer patients. Serum levels of REG4 were also found to be elevated in several cancer types and could predict metastasis and recurrence, suggesting that serum REG4 levels can potentially be used as a screening and diagnostic serum biomarker similar to carcinoembryonic antigen (CEA).

The mechanism of action of *REG4* in human cancers is complex and involves multiple pathways (**Table 2**). *REG4* is upregulated in cancer stem cells and participates in the promotion of colorectal stem cell properties *via* the Wnt/*β*-catenin pathway. The REG4/Akt/GSK-3*β*/*β*-catenin/TCF-4 pathway was also shown to regulate cell cycle progression and promote colorectal cancer cell proliferation. REG4-induced EGFR/Akt phosphorylation promotes not only cancer cell proliferation directly *via* increased AP-1 transcriptional activity, but also the polarization of macrophages to M2 phenotype, changing the microenvironment to facilitate cancer cell growth and metastasis *via* activation of CREB. Additionally, REG4 can amplify its expression *via* a positive feedback consisting of EGFR, ADAM17, TGF-α, SP1, and GPR37 was identified as an interactive partner of the REG4 complex. Some other molecules such as ADAM9, microRNAs and MAPK pathways were also found to be involved in the process of *REG4* promoting cancer cell proliferation and invasion

In this article, we specifically reviewed the expression and role of REG4 in various human cancers. The mechanisms involve promoting proliferation, apoptosis-resistance, cell cycle regulation, and TAMs. However, research on *REG4* is still at a preliminary stage, and inhibition of endogenous REG4 or its downstream signaling warrants further investigation to delineate its potential and limits for cancer diagnosis and treatment.

## Author Contributions

JZ and ZW searched PubMed about REG4 in human cancers and wrote the draft ZM and XH summarized the different functions in various human cancers ZS and HX searched and classified the complex mechanisms JZ drew the figures attached. All authors contributed to the article and approved the submitted version.

## Funding

This work was supported by a grant from the National Natural Science Foundation of China (No 81502101).

## Conflict of Interest

The authors declare that the research was conducted in the absence of any commercial or financial relationships that could be construed as a potential conflict of interest.
